# Resveratrol inhibits the phosphatidylinositide 3-kinase/protein kinase B/mammalian target of rapamycin signaling pathway in the human chronic myeloid leukemia K562 cell line

**DOI:** 10.3892/ol.2014.2014

**Published:** 2014-03-28

**Authors:** TAO SUI, LI MA, XUE BAI, QING LI, XINNV XU

**Affiliations:** 1 Department of Hematology, Tianjin First Center Hospital, Tianjin 300192, P.R. China; 2 Key Laboratory for Critical Care Medicine of the Ministry of Health, Tianjin First Center Hospital, Tianjin 300192, P.R. China

**Keywords:** phosphatidylinositide 3-kinase, protein kinase B, mammalian target of rapamycin, chronic myeloid leukemia, resveratrol

## Abstract

Resveratrol inhibits the initiation, promotion and progression of tumors, however, the mechanism by which resveratrol inhibits the proliferation of the human chronic myeloid leukemia K562 cell line remains unclear. The present study was conducted to investigate the effect of resveratrol on the activation of the phosphatidylinositide 3-kinase (PI3K)/protein kinase B (Akt)/mammalian target of rapamycin (mTOR) signaling cascade in K562 cells. Resveratrol showed significant cytotoxic effects and induced apoptosis in K562 cells in a dose- and time-dependent manner. In addition, resveratrol attenuated the phosphorylation of PI3K, Akt and mTOR in the K562 cells. Furthermore, the selected inhibitors of PI3K (LY294002), Akt (SH-6) and mTOR (rapamycin) enhanced the effects of resveratrol in K562 cells. In addition, cyclin D1 levels were found to decrease and the activation of caspase-3 was observed. Resveratrol was also found to significantly attenuate the phosphorylation of the downstream molecules, p70S6K and 4EBP1. These results suggested that the downregulation of the PI3K/Akt/mTOR signaling cascades may be a crucial mediator in the inhibition of proliferation and induction of apoptosis by resveratrol in K562 cells.

## Introduction

Chronic myeloid leukemia (CML) is a clonal myeloproliferative hematopoietic stem cell disease, characterized by the presence of the Philadelphia chromosome, which is generated by the reciprocal translocation of the ABL1 oncogene localized on chromosome 9 with the breakpoint cluster region (BCR) on chromosome 22 [t (9; 22)] (1,2). The BCL-ABL fusion gene, which has been identified as a crucial step in the pathogenesis of CML, encodes the fusion BCR-ABL1 protein, which possesses constitutive tyrosine kinase activity (3,4). The BCR-ABL tyrosine kinase significantly influences several cellular signaling cascades, including the Ras, mitogen-activated protein kinase, phosphatidylinositide 3-kinase (PI3K)/protein kinase B (Akt), signal transducer and activator of transcription 5, and Src (5–9). Abnormal overactivation of these signaling cascades leads to increased cellular proliferation, resistance to apoptosis and genetic instability.

The selective tyrosine kinase inhibitors (TKI), which target the ATP binding site of BCR/ABL, block BCR/ABL kinase activity and exhibit a positive therapeutic effect for CML (10,11). TKIs, such as imatinib, are considered as a conventional treatment option for CML. However, CML patients may become relatively resistant to TKI therapy, including the second generation TKIs (12,13) and, thus, the development of a therapeutic strategy that targets abnormal signaling cascades other than BCR/ABL is urgently required for CML treatment.

Resveratrol is a naturally occurring polyphenolic compound that is abundant in various plants, including grapes and nuts, as well as red wine and other plant-based food sources (14). Resveratrol has been reported to exert multiple beneficial effects, including anti-inflammatory, -oxidant and -viral effects, as well as neuroprotection (15,16). Resveratrol inhibits the initiation, promotion and progression of tumors and, therefore, based on the above report, resveratrol may present a potential preventive and therapeutic agent (17). Numerous studies have demonstrated that resveratrol is involved in the regulation of cell cycle arrest, differentiation and apoptosis of different tumor cell lines and experimental cancer models (18–22).

Resveratrol causes cell cycle arrest at the G1 phase by inducing the expression of the cyclin-dependent kinase inhibitors, p21WAF1/CIP1 and p27KIP1 (23). Additionally, resveratrol induces cell apoptosis by upregulating the expression of Bax, Bak, Bim, p53, tumor necrosis factor-related apoptosis-inducing ligand (TRAIL), TRAIL-receptor (R)1/R4 and TRAIL-R2/R5, whilst simultaneously downregulating the expression of B-cell lymphoma (Bcl)-2 and Bcl-XL (24,25). Numerous studies have shown that resveratrol modulates several cell signaling pathways, which are involved in the proliferation of tumor cells. Furthermore, resveratrol functions through different mechanisms in different types of tumor cells (26).

The aim of the present study was to investigate whether resveratrol has potential antitumor effects in human CML and to determine whether the modulation of the PI3K/Akt/mammalian target of rapamycin (mTOR) signaling pathway by resveratrol is crucial for its anticancer effects in the human CML K562 cell line.

## Materials and methods

### Reagents and antibodies

Resveratrol was purchased from Sigma-Aldrich (St. Louis, MO, USA) and dissolved in dimethylsulfoxide (Sigma-Aldrich) as a stock solution of 100 mM. Resveratrol was further diluted in RPMI-1640 medium (Gibco, Big Cabin, OK, USA) plus 10% fetal bovine serum (FBS; Gibco) to the appropriate final concentrations. The primary polyclonal rabbit anti-human antibodies, anti-PI3K, -phosphorylated (p)-PI3K (Tyr458), -Akt, -p-Akt (Ser473), -mTOR, -p-mTOR (Ser2448), -p70S6K, -p-p70S6K (Thr389), -4EBP1, -p-4EBP1 (Ser65), -cyclin D1, -procaspase-3, -cleaved caspase-3 and-β-actin, were obtained from Cell Signaling Technology, Inc. (Beverly, MA, USA), and the secondary horseradish peroxidase (HRP)-labeled mouse anti-rabbit IgG polyclonal antibodies for western blot analysis were provided by Beijing Zhongshan Golden Bridge Biotechnology Co., Ltd. (Beijing, China). Annexin V-fluorescein isothiocyanate (FITC) and propidium iodide (PI) were purchased from BD Biosciences (Palo Alto, CA, USA), LY294002 and SH-6 were provided by Santa Cruz Biotechnology, Inc. (Dallas, TX, USA), and rapamycin (RAPA) was purchased from the North China Pharmaceutical Group Corporation (Shijiazhuang, China).

### Cell culture

The human CML K562 cell line was purchased from the Peking Union Medical College Cell Library (Beijing, China). The cells were cultured in RPMI-1640 medium supplemented with 100 U/ml of penicillin, 100 μg streptomycin (both Gibco) and 10% FBS at 37°C in a humidified atmosphere containing 5% CO_2_.

### Cell Counting Kit-8 (CCK-8) assay

Cell proliferation was determined using water-soluble tetrazolium salt-8 dye (Sigma-Aldrich) according to the manufacturer’s instructions. Briefly, the cells were suspended in RPMI-1640 medium containing 10% FBS and seeded at a density of 5×10^3^ cells/well in 96-well plates. Resveratrol was then added to the medium at various concentrations of up to 60 μM for different time durations. Next, the CCK-8 solution (10 μl; Sigma-Aldrich) was added to each well and further incubated at 37°C for 3 h. The absorbance was determined at a wavelength of 450 nm using a microplate reader (Bio-Rad, Hercules, CA, USA). In total, three duplicate wells were set up for each experimental condition.

### Apoptosis analysis

A total of 1×10^6^ cells/well were treated with resveratrol for 24 h and double-staining with Annexin V-FITC (1 μl) and PI (1 μg) was performed. The cells were then washed with phosphate-buffered saline (PBS; Beyotime, Haikou, China) and analyzed by flow cytometry (FACS sort; BD Biosciences).

### Western blot analysis

Western blot analysis was performed on whole cell extracts obtained by the direct dissolution of cells using a whole cell protein extract reagent (M-PER; Pierce Biotechnology, Inc., Rockford, IL, USA) according to the manufacturer’s instructions. Protein concentrations were then determined using a bicinchoninic acid protein assay kit and bovine serum albumin [both Sangon Biotech (Shanghai) Co,. Ltd., Shanghai, China] was used as a control. Next, the proteins (40 μg/lane) were separated on 12% SDS-PAGE gels (Beyotime) and transferred onto polyvinylidene difluoride membranes (Bio-Rad). The membranes were then blocked with 5% non-fat milk in phospshate-buffered saline with Tween [PBST; 0.2% Tween-20 in PBS (pH 7.6); Beyotime] and incubated with the primary antibodies (1:1,000) for 18–24 h at 4°C. The membranes were subsequently incubated with the secondary antibodies conjugated to HRP (1:5,000) for 1 h at 37°C. Finally, the protein bands were visualized using an enhanced chemiluminescence western blot detection kit (Pierce Biotechnology, Inc.).

### Statistical analysis

Data are presented as the mean ± standard deviation and were analyzed using SPSS 16.0 software (SPSS, Inc., Chicago, IL, USA). Significant differences were determined using one-way analysis of variance or a two-tailed Student’s t-test. P<0.05 was considered to indicate a statistically significant difference.

## Results

### Resveratrol inhibits the proliferation and induces the apoptosis of K562 cells

**Figure 1:**
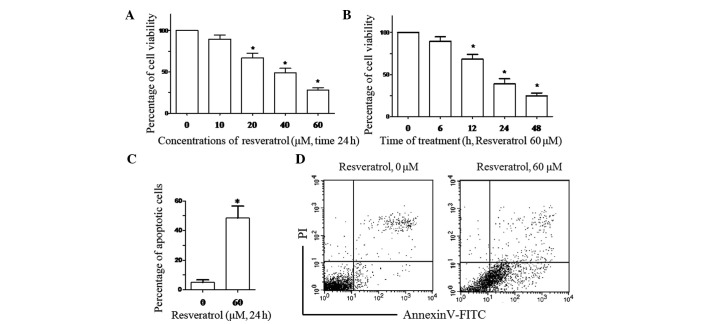
Resveratrol inhibits proliferation and induces apoptosis in K562 cells. Cells were treated at the indicated times and concentrations. The percentage of cell viability was determined by Cell Counting Kit-8 assay and resveratrol inhibited growth activity in a (A) dose- and (B) time-dependent manner. (C) Effect of resveratrol on apoptosis. (D) Double-staining with Annexin V-FITC and PI analyzed by flow cytometric following the exposure of cells to resveratrol for 24 h is presented as a representative plot of double staining with Annexin V-FITC and PI. Data are expressed as the mean ± standard deviation of triplicate experiments.^*^P<0.05 vs. 0 μM resveratrol treatment. FITC, fluorescein isothiocyanate; PI, propidium iodide.

To investigate the effect of resveratrol on the proliferation of K562 cells, the cells were treated with resveratrol at serial concentrations and its ability to inhibit proliferation was measured using a CCK-8 assay. The results showed that resveratrol may inhibit the proliferation of K562 cells in a dose- and time-dependent manner (Fig. 1A and B). To further investigate whether resveratrol can induce the apoptosis of K562 cells, the cells were treated with 60 μM of resveratrol for 24 h and the apoptotic rate of the K562 cells was detected using Annexin V-FITC/PI staining (Fig. 1C and D). The results suggested that resveratrol inhibits cell proliferation and induces apoptosis in K562 human CML cells.

### Resveratrol blocks PI3K/Akt phosphorylation in K562 cells

**Figure 2:**
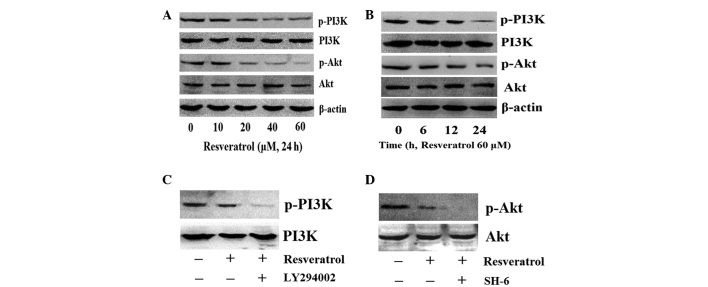
Resveratrol reduces the phosphorylation of PI3K and Akt in K562 cells. Levels of p-PI3K and p-Akt in K562 cells treated with (A) various concentrations of resveratrol for 24 h and (B) 60 μM of resveratrol for different time periods, as determined by western blot analysis. (C) The selective inhibitor, LY294002, enhanced the resveratrol effect on reducing the phosphorylation of PI3K and (D) the selective inhibitor, SH-6, enhanced the resveratrol effect on reducing the phosphorylation of Akt. β-actin was used as a loading control for PI3K and Akt, while PI3K and Akt were used as loading controls for p-PI3K and Akt, respectively. PI3K, phosphatidylinositide 3-kinase; p-PI3K, phosphorylated-PI3K; Akt, protein kinase B; p-Akt, phosphorylated-Akt.

The PI3K/Akt signaling pathway is important in cell proliferation, differentiation and survival and resveratrol has been demonstrated to inhibit proliferation and induce apoptosis in various types of cancer cells (27). In the present study, to investigate whether PI3K/Akt phosphorylation is responsible for resveratrol-induced inhibition of proliferation, the K562 cells were treated with various concentrations of resveratrol for 24 h. The levels of p-PI3K and p-Akt were then detected and the results demonstrated that resveratrol significantly blocks the constitutive phosphorylation of PI3K at Tyr458 and Akt at Ser473 in a dose-dependent manner (Fig. 2A). The K562 cells were then treated with 60 μM of resveratrol for 0, 6, 12 and 24 h, and the levels of p-PI3K and p-Akt were observed to decrease following 24 h of treatment (Fig. 2B). The K562 cells were also treated with the selective PI3K inhibitor, LY294002, or the selective Akt inhibitor, SH-6. The selective inhibitors were observed to further inhibit the phosphorylation of PI3K and Akt in K562 cells induced by resveratrol, respectively (Fig. 2C). These results indicated that the antiproliferative effects of resveratrol in K562 cells are associated with blocking the activation of the PI3K/Akt signaling cascade.

### Resveratrol suppresses the phosphorylation of mTOR in K562 cells

**Figure 3:**
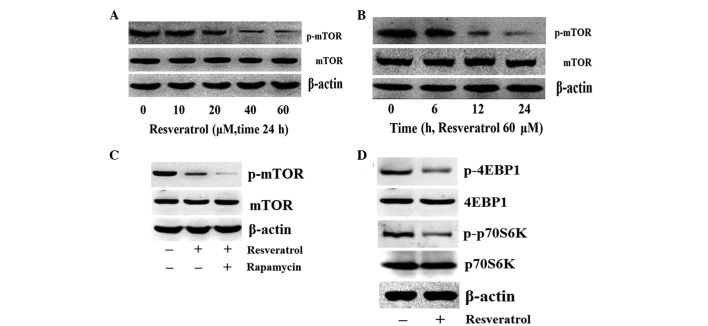
Resveratrol suppresses the phosphorylation of mTOR and its downstream targets in K562 cells. Levels of p-mTOR in K562 cells treated with (A) various concentrations of resveratrol for 24 h (which were then lysed) and (B) 60 μM of resveratrol for different time periods, as determined by western blot analysis. (C) Inhibitor rapamycin enhanced the resveratrol effect on reducing the phosphorylation of mTOR and (D) resveratrol reduced the phosphorylation of p70S6K and 4EBP1. β-actin was used as a loading control for mTOR, 4EBP1 and p70S6k, while mTOR, 4EBP1 and p70S6k were used as loading controls for p-mTOR, p-4EBP1 and p-p70S6K, respectively. mTOR, mammalian target of rapamycin; p-mTOR, phosphorylated-mTOR.

The mTOR protein is one of the downstream targets of PI3K/Akt. To investigate the effect of resveratrol on mTOR phosphorylation, the K562 cells were treated with a serial concentration of resveratrol for 24 h and resveratrol was found to reduce the levels of p-mTOR (Ser2448) in a dose-dependent manner (Fig. 3A). The K562 cells were then treated with 60 μM of resveratrol for 0, 6, 12 and 24 h and the level of p-mTOR was observed to decrease following resveratrol treatment for 12 h (Fig. 3B), and was further reduced following rapamycin treatment (Fig. 3C). These results indicated that resveratrol inhibits the PI3K/Akt/mTOR signaling pathway and that the specific inhibitor of mTOR enhances the effect induced by resveratrol.

Next, the effect of resveratrol on the downstream targets of mTOR was investigated. The K562 cells were treated with 0 or 60 μM of resveratrol for 24 h and the downstream targets were detected using western blot analysis. The results revealed that treatment with resveratrol reduced the phosphorylation of the downstream targets, p70S6K and 4EBP1; however, total p706SK and 4EBP1 levels were not affected by resveratrol treatment (Fig. 3D), which indicated that resveratrol may downregulate the Akt/mTOR signaling pathway.

### Resveratrol suppresses cyclin D1 and enhances caspase-3 expression

**Figure 4:**
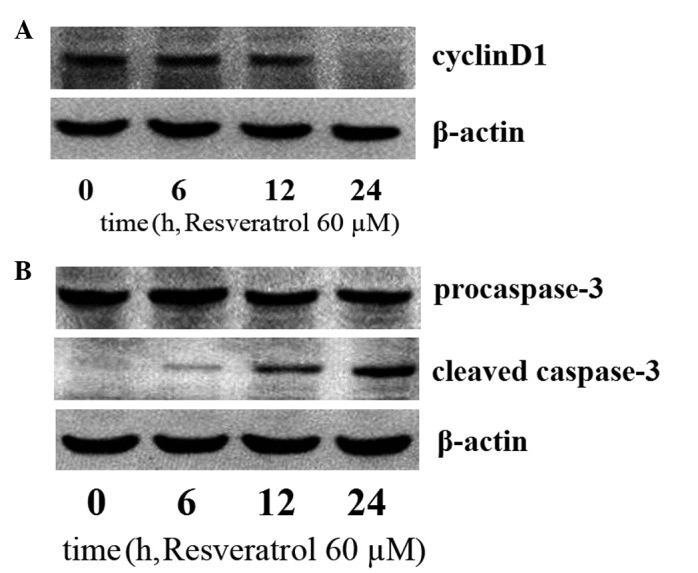
Resveratrol decreases cyclin D1 and increases caspase-3 expression. The expression of (A) cyclin D1 and (B) pro- and cleaved-caspase-3 was detected by western blot analysis in K562 cells which were treated with 60 μM of resveratrol for different time periods. β-actin was used as a loading control for cyclin D1 and procaspase-3, while procaspase-3 was used as a loading control for cleaved caspase-3.

Constitutive activation of the PI3K/Akt/mTOR signaling pathway has been demonstrated to exhibit a critical function in the cell cycle and antiapoptosis by affecting several regulatory molecules, including the upregulation of cyclin D1 and downregulation of caspase-3 expression (28). In the current study, to investigate whether resveratrol downregulates cyclin D1 expression and upregulates caspase-3 expression, the K562 cells were treated with 60 μM of resveratrol. A marked decline in cyclin D1 levels and an increase in caspase-3 levels in the resveratrol-treated cells was observed (Fig. 4).

## Discussion

Although resveratrol has been reported to have a wide range of potential targets during the inhibition of proliferation and induction of apoptosis in a variety tumor cell types (28), the underlying molecular mechanisms of its anticancer effects are not well understood, particularly in human leukemia which often proves difficult to treat as multiple signaling pathways may be involved (17). Contradictory results have previously been reported with regard to the inhibition of proliferation and induction of apoptosis by resveratrol. Certain studies have reported that resveratrol treatment induces apoptosis in various tumor cells (29–33). However, other studies have reported that resveratrol induces differentiation, but not apoptosis, in certain types of cancer cells (34–36). In the current study, one of the possible mechanisms of resveratrol-induced apoptosis of human CML K562 cells, was investigated.

There is much evidence to support the critical function of the PI3K/Akt/mTOR signaling pathway in cancer proliferation, tumor genesis and metastasis (37,38), and numerous studies have demonstrated that PI3K/Akt/mTOR activity is increased in a variety of tumor cell lines (39–42), including leukemia (43). In addition, it has been demonstrated that the inhibition of PI3K/Akt activation or expression may inhibit cancer cell proliferation and invasion. This signaling pathway involves three key driving proteins, PI3K, Akt and mTOR. The PI3K proteins are a family of lipid kinases, which include PI3K1, PI3K2 and PI3K3. Akt belongs to a family of serine/threonine protein kinases, which can be activated in a PI3K-dependent manner following stimulation by growth factors, stress or protein phosphatases. Furthermore, mTOR is one of the main downstream target molecules of PI3K/Akt, which performs a crucial function in the regulation of proliferation, differentiation and survival of cells. It has been demonstrated that the activation of the PI3K/Akt/mTOR signaling pathway increases the phosphorylation of p70S6K and 4EBP1, and that p-p70S6K subsequently induces the phosphorylation of the ribosomal protein S6.

The effect of resveratrol on the PI3K/Akt/mTOR pathway in CML cells has not been widely investigated. The results of the current study revealed that resveratrol inhibits the proliferation and induces the apoptosis of K562 cells in a dose-dependent manner. In addition, resveratrol downregulates the PI3K/Akt/mTOR signaling pathway and the inhibitors of the proteins involved in this signaling pathway enhance the inhibitory effects induced by resveratrol in K562 cells. Furthermore, resveratrol inhibits proliferation and induces apoptosis in human leukemia K562 cells, which is considered to occur via the deregulation of the cell cycle machinery and activation of mitochondria-mediated caspase-3 dependent apoptotic signaling cascades. These results indicated that resveratrol is an attractive candidate for use in leukemia therapy. Therefore, further understanding of the underlying molecular signaling mechanisms of the inhibition of proliferation and induction of CML cell apoptosis induced by resveratrol may aid the development of additional therapeutic targets for the treatment of CML.

Resveratrol inhibits the proliferation and induces the apoptosis of cells mediated by the regulation of cell cycle, proapoptotic and antiapoptotic proteins, such as cyclin D1 and caspase-3. Gao *et al* (44) demonstrated that the PI3K/Akt/mTOR signaling pathway exhibits a key function in the cell cycle progression in human prostate cancer cells. In addition, resveratrol has been demonstrated to inhibit the proliferation and induce the apoptosis of cells by downregulation of the nuclear factor-κβ signaling pathway (45).

In conclusion, the present study demonstrated that resveratrol downregulates and inactivates the PI3K/Akt/mTOR signaling pathway, which may exhibit a critical function in resveratrol-induced apoptosis in K562 cells and, therefore, the PI3K/Akt/mTOR signaling pathway may present a potential therapeutic target for the treatment of human CML.
